# Prognostic value of cervical nodal necrosis on staging imaging of nasopharyngeal carcinoma in era of intensity-modulated radiotherapy: a systematic review and meta-analysis

**DOI:** 10.1186/s40644-022-00462-6

**Published:** 2022-05-20

**Authors:** Qi-Yong H. Ai, Kuo Feng Hung, Tiffany Y. So, Frankie K. F. Mo, Wing Tsung Anthony Chin, Edwin P. Hui, Brigette B. Y. Ma, Michael Ying, Ann D. King

**Affiliations:** 1 Department of Health Technology and Informatics, The Hong Kong Polytechnic University, Hung Hom, Kowloon, Hong Kong S.A.R. P.R. China; 2 Department of Imaging and Interventional Radiology, The Chinese University of Hong Kong, Prince of Wales Hospital, New Territories, Hong Kong S.A.R. P.R. China; 3 Division of Oral and Maxillofacial Surgery, Faculty of Dentistry, University of Hong Kong, Hong Kong, Hong Kong S.A.R. P.R. China; 4 Department of Clinical Oncology, State Key Laboratory in Oncology in South China, The Chinese University of Hong Kong, Prince of Wales Hospital, Sir Y.K. Pao Centre for Cancer, New Territories, Hong Kong S.A.R. P.R. China; 5 Department of Radiology and Organ Imaging, United Christian Hospital, Kowloon, Hong Kong S.A.R. P.R. China

**Keywords:** Nasopharyngeal carcinoma, Cervical nodal necrosis, Imaging, Prognostic value, Meta-analysis

## Abstract

**Purposes:**

To systematically review and perform meta-analysis to evaluate the prognostic value of cervical nodal necrosis (CNN) on the staging computed tomography/magnetic resonance imaging (MRI) of nasopharyngeal carcinoma (NPC) in era of intensity-modulated radiotherapy.

**Methods:**

Literature search through PubMed, EMBASE, and Cochrane Library was conducted. The hazard ratios (HRs) with 95% confidence intervals (CIs) of CNN for distant metastasis-free survival (DMFS), disease free survival (DFS) and overall survival (OS) were extracted from the eligible studies and meta-analysis was performed to evaluate the pooled HRs with 95%CI.

**Results:**

Nine studies, which investigated the prognostic values of 6 CNN patterns on MRI were included. Six/9 studies were eligible for meta-analysis, which investigated the CNN presence/absence in any nodal group among 4359 patients. The pooled unadjusted HRs showed that the CNN presence predicted poor DMFS (HR =1.89, 95%CI =1.72-2.08), DFS (HR =1.57, 95%CI =1.08-2.26), and OS (HR =1.87, 95%CI =1.69-2.06). The pooled adjusted HRs also showed the consistent results for DMFS (HR =1.34, 95%CI =1.17-1.54), DFS (HR =1.30, 95%CI =1.08-1.56), and OS (HR =1.61, 95%CI =1.27-2.04). Results shown in the other studies analysing different CNN patterns indicated the high grade of CNN predicted poor outcome, but meta-analysis was unable to perform because of the heterogeneity of the analysed CNN patterns.

**Conclusion:**

The CNN observed on the staging MRI is a negative factor for NPC outcome, suggesting that the inclusion of CNN is important in the future survival analysis. However, whether and how should CNN be included in the staging system warrant further evaluation.

## Keypoints

1. Cervical nodal necrosis (CNN) is frequently observed in pre-treatment nasopharyngeal carcinoma (NPC);
2. CNN on pre-treatment imaging of NPC is a negative prognosticator of outcome.
3. Inclusion of CNN is critical in the future survival analysis in NPC;
4. It remains unclear whether/how should CNN be included in the staging system.

## Introduction

Nasopharyngeal carcinoma (NPC) is a cancer that derives from the epithelial tissues at the back of the nose and prone to spread outside to the adjacent cervical lymph nodes. About 70% of patients have metastatic lymph nodes at presentation [1], often at multiple levels in one or both sides of the neck, and these nodes are an important determinant of survival outcome. Imaging, such as computed tomography (CT) and magnetic resonance imaging (MRI), has advantages over clinical examinations in depicting this disease, and has been widely used to evaluate the regional metastatic nodes at presentation [2, 3]. Cervical nodal necrosis (CNN) is one of the nodal characteristics frequently observed in metastatic nodes by CT and MRI [4–6]. Necrosis is usually associated with tissue hypoxia, which is a strong negative factor for outcome [7, 8] , and so the CNN may have potential to predict outcome. Indeed, some previous studies showed the presence of CNN predicted poor outcome in NPC, but conflicting results also have reported [9–27].

To better understand the prognostic value of the CNN in patients with NPC, this review systematically evaluated and summarised the existing literature on the prognostic value of CNN observed on the staging CT/MRI in patients with NPC treated with intensity-modulated radiotherapy (IMRT). Meta-analysis was performed for the data extracted from the eligible studies. This information may clarify the prognostic value of CNN and help to identify the role of CNN in the cancer staging system.

## Methods

### Search strategy

Methods of the analysis and inclusion criteria were defined in advance, documented, and the proposal was registered at PROSPERO International Prospective Register of Systematic Reviews (ID: CRD42021286561). This study followed the Meta-analyses of Observational Studies in Epidemiology Guidelines [28]. Studies published from the onset of electronic databases up to September 2021 in the PubMed, EMBASE, and Cochrane Library databases were searched with the keywords of “nasopharyngeal AND (carcinoma OR cancer) AND (necrotic OR necrosis) AND (Magnetic Resonance Imaging OR mri OR computed tomography OR CT)”. Records were collated Endnote^TM^ (Version: X7, Clarivate Analytics, New York, NY, USA), and the titles were screened for duplicates. An Institutional Review Board approval or patient written consent was waived as we only used data from the previously published studies.

### Selection criteria

The inclusion criteria were as follows:

(1) Original articles published in English;

(2) Participants: studies with patients with biopsy-proven NPC without distant metastasis and patients who underwent the pre-treatment head and neck staging CT or MR imaging scans;

(3) Comparison: studies that evaluated CNN using CT or MRI;

(4) Outcome: distant metastases-free survival (DMFS) and disease-free survival (DFS) or overall survival (OS).

The exclusion criteria were as follows:

(1) Articles in conference abstracts, case reports, review, and letter to editor formats;

(2) Studies with patients who underwent other than IMRT;

(3) Studies not reporting unadjusted or adjusted HR and 95%CI HR of CNN for DMFS; and

(4) Studies with the patient population overlapped with the previous studies conducted in the same investigated institution for assessing the same CNN pattern.

The records were independently selected by two observers (QYHA and TYS) by assessing the title and then abstracts for the full-text evaluation. Any discrepancy at these steps was resolved by discussion. An additional manual search was performed in the reference lists of the included studies.

### Data extraction

Data extraction was performed independently by two observers (QYHA and KFH), and the following data were retrieved: first author, journal name, year of publication, city, hospital/institution, patient recruitment period, number of patients, patient treated with chemotherapy, patients with nodes, patients with CNN, age, sex, follow-up time, and edition of the AJCC staging system for NPC, histological type, CNN patterns, group of patients for survival analysis, unadjusted and adjusted HRs with 95% CI of CNN for the survival endpoints. When HRs with 95% CIs for outcome endpoints were not provided, we used the methods described by Tierney et al. [29] to estimate them from the data extracted from the respective Kaplan–Meier survival curves using the WebPlotDigitizer [30]. Necrosis was identified as a focal area of high signal intensity on T2-weighted MRI, as an area of non-enhancement on contrast-enhanced T1-weighted MRI, or as ﻿an area of low attenuation with or without a surrounding rim of enhancement on contrast-enhanced CT [4, 5].

### Survival endpoints

DMFS was set as the primary survival endpoint for the analysis as the distant metastases is the main causes of mortality in NPC. DFS and OS were also included as the additional survival endpoints for the analysis if information was provided. DMFS, DFS and OS were calculated from the date of the diagnosis/start of treatment/end of the treatment to the date of distant metastases, date of any disease recurrence, and date of death, respectively.

### Quality assessment

The Newcastle-Ottawa-Scale (NOS) [31] for cohort studies to assess the quality of the included studies. The NOS evaluated each study in three parts. The NOS score ranges from 0 to 9, of which 9 indicates the highest quality. The quality assessment was performed by two observers (QYHA and KFH) and discrepancies were solved by discussion with a third observer (TYS).

### Statistical analysis

The inter-observer agreements for the article selection by the titles and abstracts were calculated, respectively, and the Cohen’s kappa coefficients were obtained. The meta-analysis was performed for the unadjusted and adjusted HRs of CNN for the survival endpoint(s), respectively. The HRs with 95%CI were pooled with a random- or fixed- effects models. Heterogeneity between studies was assessed using the Cochran Q-statistic and I^2^ tests. The analysis with an I^2^ value larger than 50% was considered as substantial heterogeneity. The fixed-effects model was used if the heterogeneity was not significant (I^2^ < 50% and *p*-value > 0.05); otherwise, a random-effects model was applied. To test whether a single study affected the combined HR, a sensitivity analysis was performed. All statistical tests were two-sided, and a *p*-value of less than 0.05 was considered to indicate a statistically significant difference. Publication bias was tested by Begg’s test and Egger’s test. For the interpretation of Egger’s test, statistical significance was defined as *p*-value of less than 0.1. The meta-analysis was performed using Stata IC (Version 16.0, StataCorp LLC, TX, USA).

## Results

### Study selection

**Fig. 1:**
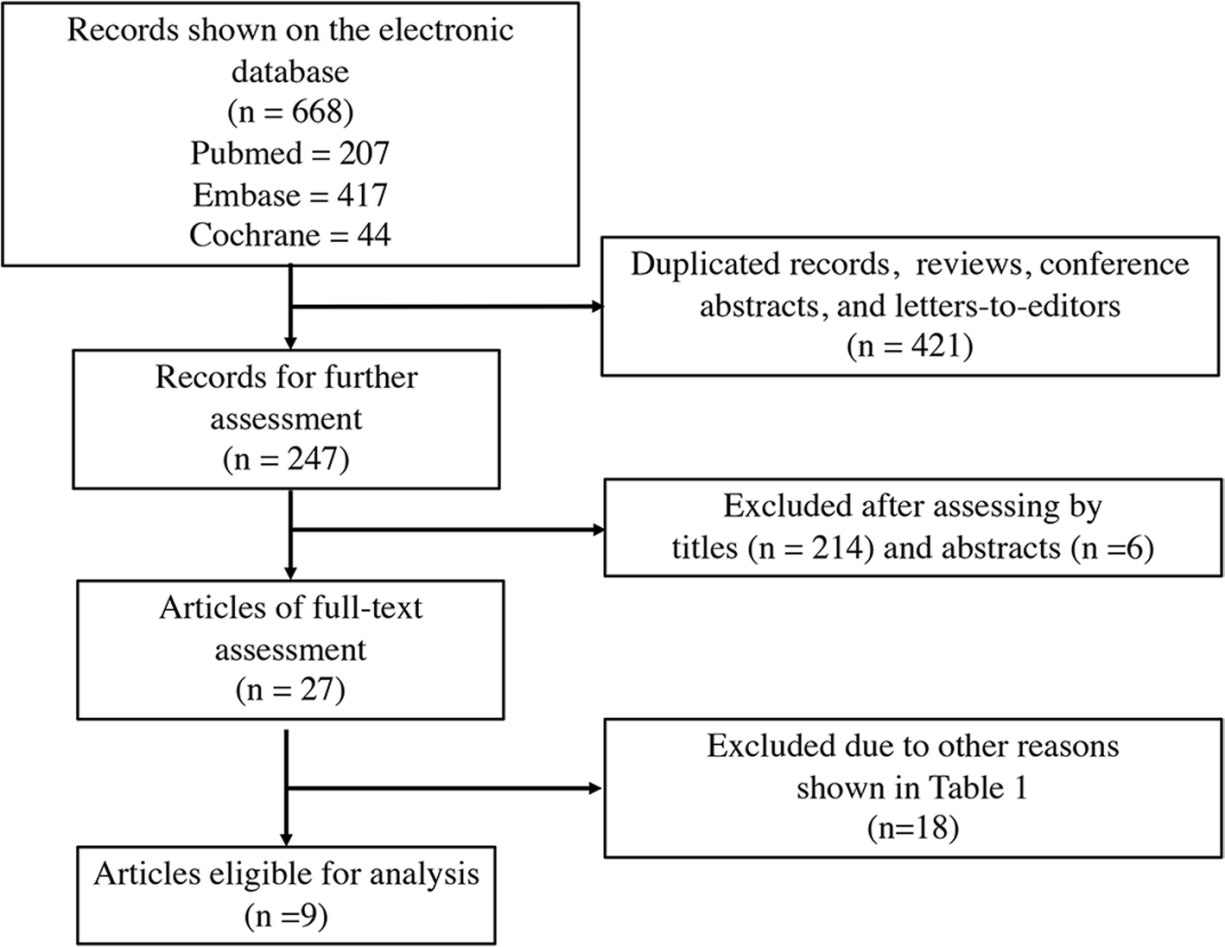
Flowchart for study selection

Figure 1 shows the flowchart for study selection. A total of 668 potential records were initially identified by searching the electronic databases (Fig. 1). After the initial exclusion of the duplicates, reviews, conference abstracts, and letters (*n* = 421), and further exclusion of 214 and 6 records after assessing the titles and abstracts, respectively, 27 studies remained for full-text reading. No additional articles were identified through manual search from the reference list of the included studies. The kappa coefficients for the study selection by assessing the titles and abstracts were 0.81 and 0.83, respectively. Eighteen studies were subsequently excluded due to the specific reasons (Table 1) [9–18, 32–39], leaving 9 studies for the further systematic review and analysis [19–27].

**Table 1:** Numbers and reasons for the excluded articles after full-text review

**Reasons to exclude the articles after the full-text review**	**Numbers of studies**	**Numbers in the references list**
Patients treated with other than IMRT	3	[9–11]
HRs of CNN for DMFS was not performed or unable to calculate	7	[12–18]
Survival analysis was not performed for CNN	2	[32, 33]
CNN + necrotic primary tumour as one variable was assessed	1	[34]
Studies conducted by the same institution with overlapping patient recruitment periods and analysis performed for the same CNN pattern	5	[35–39]

*IMRT* Intensity-modulated radiotherapy, *DMFS* Distant metastases free survival, *HR* Hazard ratio, *CNN* Cervical nodal necrosis

### Characteristics of the eligible studies

Characteristics of the included studies are shown in Table 2. The included studies were conducted by 5 institutions and the CNN were all evaluated on MRI. Three conducted by the same institution were included because these studies performed analysis to evaluate prognostic values of different CNN patterns [25–27]. Analysis was performed to evaluate prognostic values of 6 CNN patterns, which included (1) CNN presence/absence in any nodal group (*n* =6) [19–24], (2) CNN presence/absence in retropharyngeal nodes (RPNs) (*n* =1) [25], (3) CNN grades (*n* =1) [26], (4) CNN laterality (*n* =1) [27], (5) total CNN volume [23], and (6) maximum percentage of nodal necrotic volume of one single node (necrosis%) [23]. According to the NOS criteria, the quality of the eligible studies ranged from 7 to 9 with a median score of 8 (Table 3).

**Table 2:** Characteristics of the eligible articles

First author	Year of Publication	City	Patient recruitment period	Total patients	Patients treated with chemotherapy	Patients with nodes	Patients with CNN	Imaging modality	Numbers of undifferentiated + non-keratinising types	AJCC /UICC Edition	Median follow-up time in months (range)	Patient group for analysis
CNN presence/absence in any nodal group												
Li [19]	2013	Guangzhou	2003-2007	749	535	565	142	MRI	744	7th	60.7 (3-104)	N+ group
Zhang [20]	2017	Guangzhou	2009-2012	1302	1193	1302	448	MRI	1294	7th	47.8 (1.3-75.3)	N+ group
Zhou [21]	2018	Shanghai	2010-2011	354	300	320	143	MRI	353	7th	63 (Not mentioned)	All
Feng [22]	2019	Hangzhou	2007-2012	616	601	616	235	MRI	612	8th	62.6 (3.4 -119)	N+ group
Ai [23]	2019	Hong Kong	2005-2012	546	382	404	153	MRI	544	8th	82.3 (3.2-150)	N+ group
Xu [24]	2021	Xi’an	2006-2018	792	744	687	501	MRI	789	7th	46.2 (1.3-130)	All
CNN presence/absence in retropharyngeal nodes												
Tang [25]	2014	Guangzhou	2003-2007	749	535	565	64/481 (RPN+)	MRI	744	7th	81 (3-127)	N+ group
CNN grades												
Zhang [26]	2017	Guangzhou	2009-2012	1423	1310	1423	Grade 1: 213 Grade 2: 257	MRI	1415	7th	48.6 (1.3-76)	N+ group
CNN laterality												
Xie [27]	2020	Guangzhou	2010-2013	733	634	559	No/unilateral CNN: 692 Bilateral CNN: 41	MRI	728	8th	62 (1.4-83.2)	All

*CNN* Cervical nodal necrosis, *AJCC/UICC* American Joint of Cancer Committee/ Union for International Cancer Control, *N+group* Patients with metastatic nodes, *RPN* Retropharyngeal node, *MRI* Magnetic resonance imaging

**Table 3:** The Newcastle-Ottawa Scale (NOS) quality assessment of the eligible studies in the meta-analysis

First author	Year of publication	Selection				Comparability	Outcome			Total score
		Representativeness of the exposed cohort (0 - 1)	Selection of the non-exposed cohort (0 -1)	Ascertainment of exposure (0 -1)	Demonstration that outcome of interest was not present at start of study (0 -1)	Comparability of cohorts on the basis of the design or analysis (0 -2)	Assessment of outcome (0 -1)	Was follow-up long enough for outcomes to occur (0 -1)	Adequacy of follow up of cohorts (0 -1)	
CNN presence/absence in any nodal group										
Li [19]	2013	1	1	0	1	2	1	1	1	8
Zhang [20]	2017	1	1	1	1	2	0	1	1	8
Zhou [21]	2018	1	1	1	1	2	0	1	1	8
Feng [22]	2019	1	1	1	1	2	0	1	1	8
Ai [23]	2019	1	1	1	1	2	1	1	1	9
Xu [24]	2021	1	1	0	1	2	0	1	1	7
CNN presence/absence in retropharyngeal nodes										
Tang [25]	2020	1	1	0	1	2	1	1	1	8
CNN grades										
Zhang [26]	2021	1	1	1	1	2	1	1	1	9
CNN laterality										
Xie [27]	2021	1	1	1	1	2	1	1	1	9

Three studies were excluded from the meta-analysis due to the great heterogeneity of the analysed CNN patterns or the limited studies for evaluating each of the CNN patterns. Meta-analysis therefore was only eligible to perform in 6 studies which evaluated the prognostic value of CNN presence/absence in any nodal group [19–24]. The eligible studies for meta-analysis were published over 9 years (2013 [19]– 2021 [24]) (Table 2). Patient recruitment ranged from 2003 to 2018 with the follow-up periods ranged from 1.3 to 150 months (Table 2). The total patient number extracted from these studies was 4359 (range 354 –1302) with ages ranging from 12 to 90 years, of which 3216 (73.8%) were male and 1143 (26.2%) were female. Metastatic cervical nodes were observed in 3894 patients, of which CNN was observed in 1622/3894 (41.7%) patients (Table 2). Over 99% of patients had undifferentiated carcinoma or non-keratinising carcinoma (Table 2).

### Prognostic value of the CNN presence/absence

**Fig. 2:**
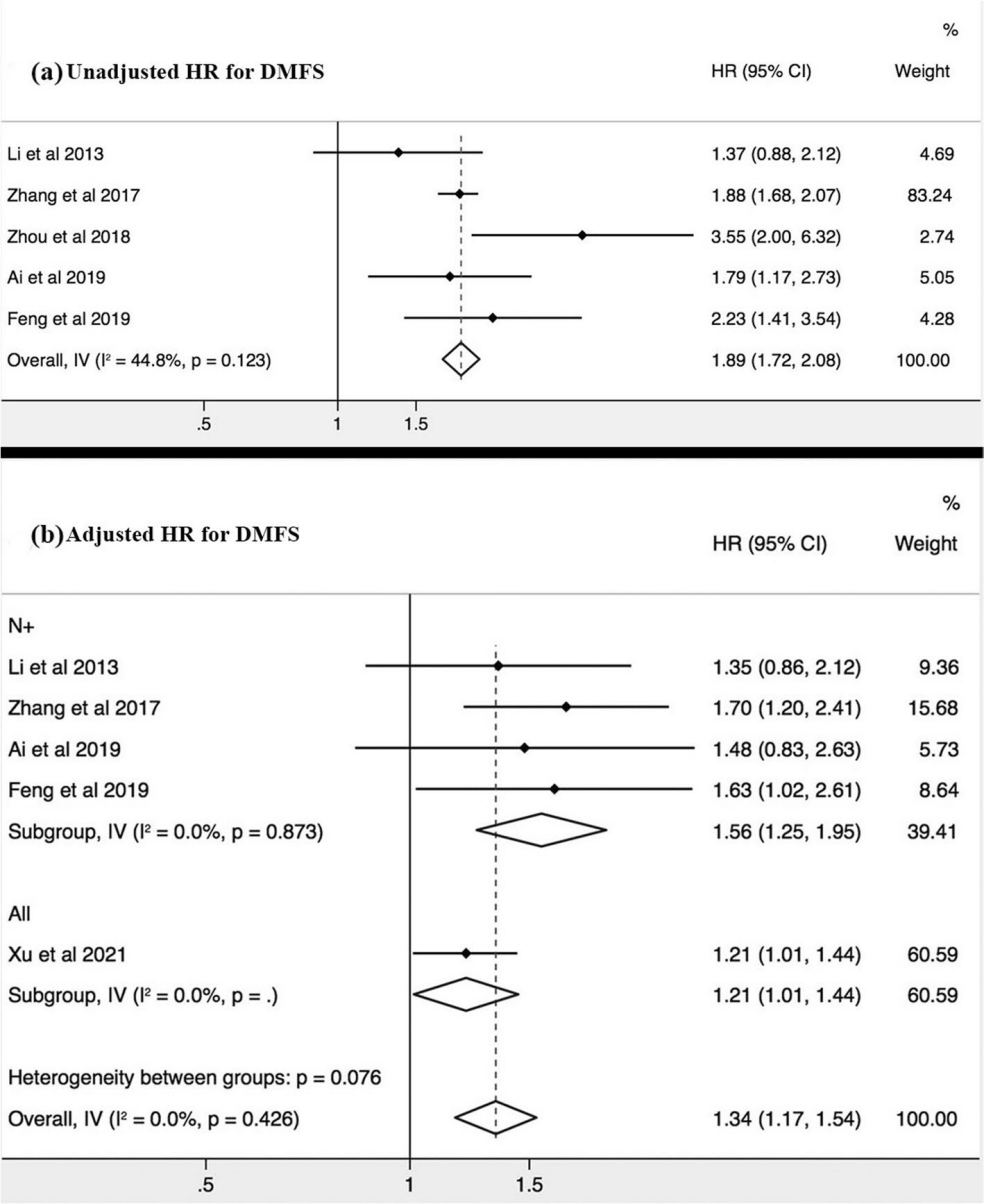
Forest plots of the meta-analysis showing the pooled hazard ratios (HRs) of CNN presence in any nodal group for distant metastases free survival (DMFS). Meta-analysis was performed for the unadjusted HRs (**a**) and adjusted HRs (**b**), respectively

**Fig. 3:**
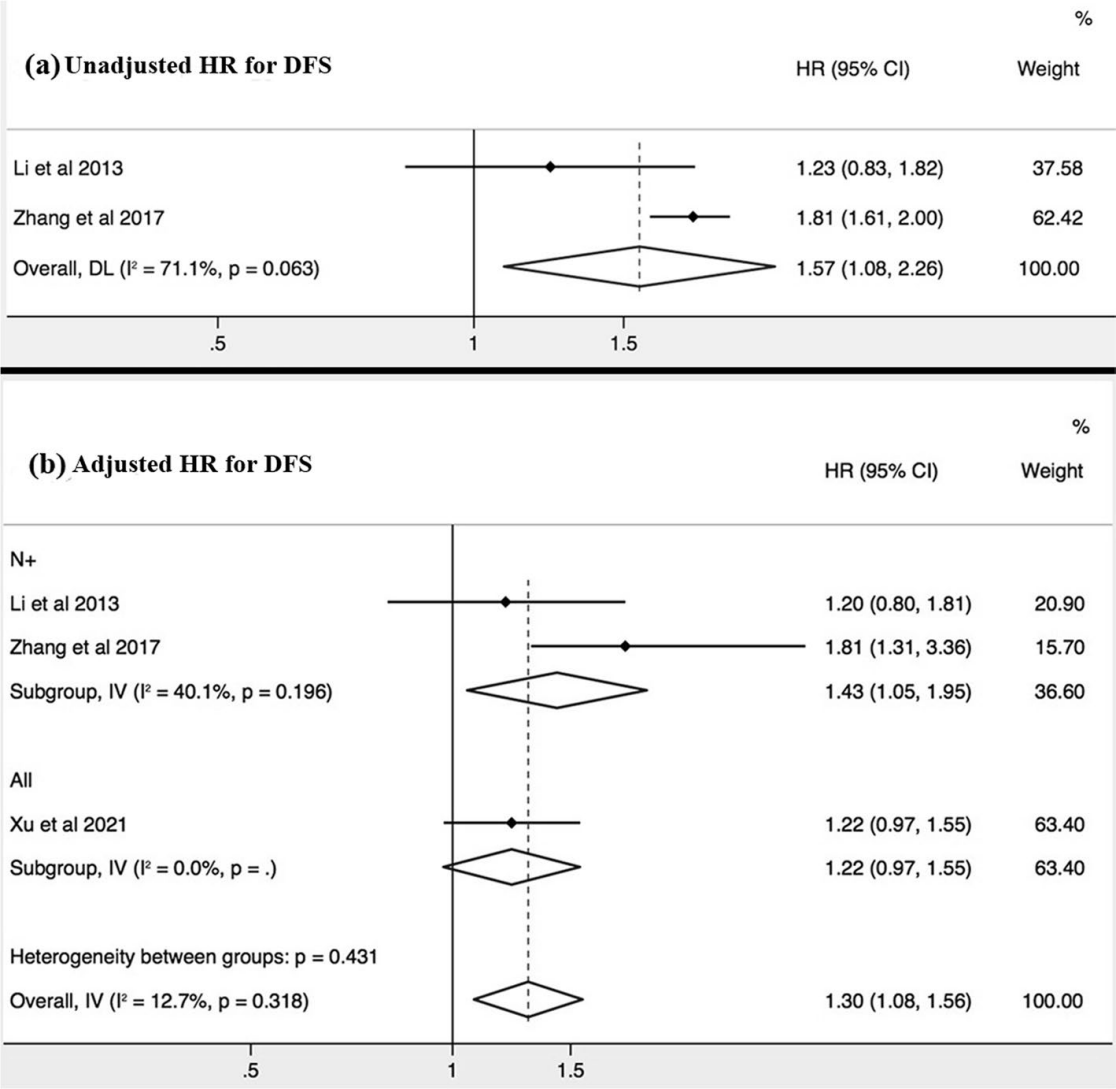
Forest plots of the meta-analysis showing the pooled hazard ratios (HRs) of CNN presence in any nodal group for disease free survival (DFS). Meta-analysis was performed for the unadjusted HRs (**a**) and adjusted HRs (**b**), respectively

**Fig. 4:**
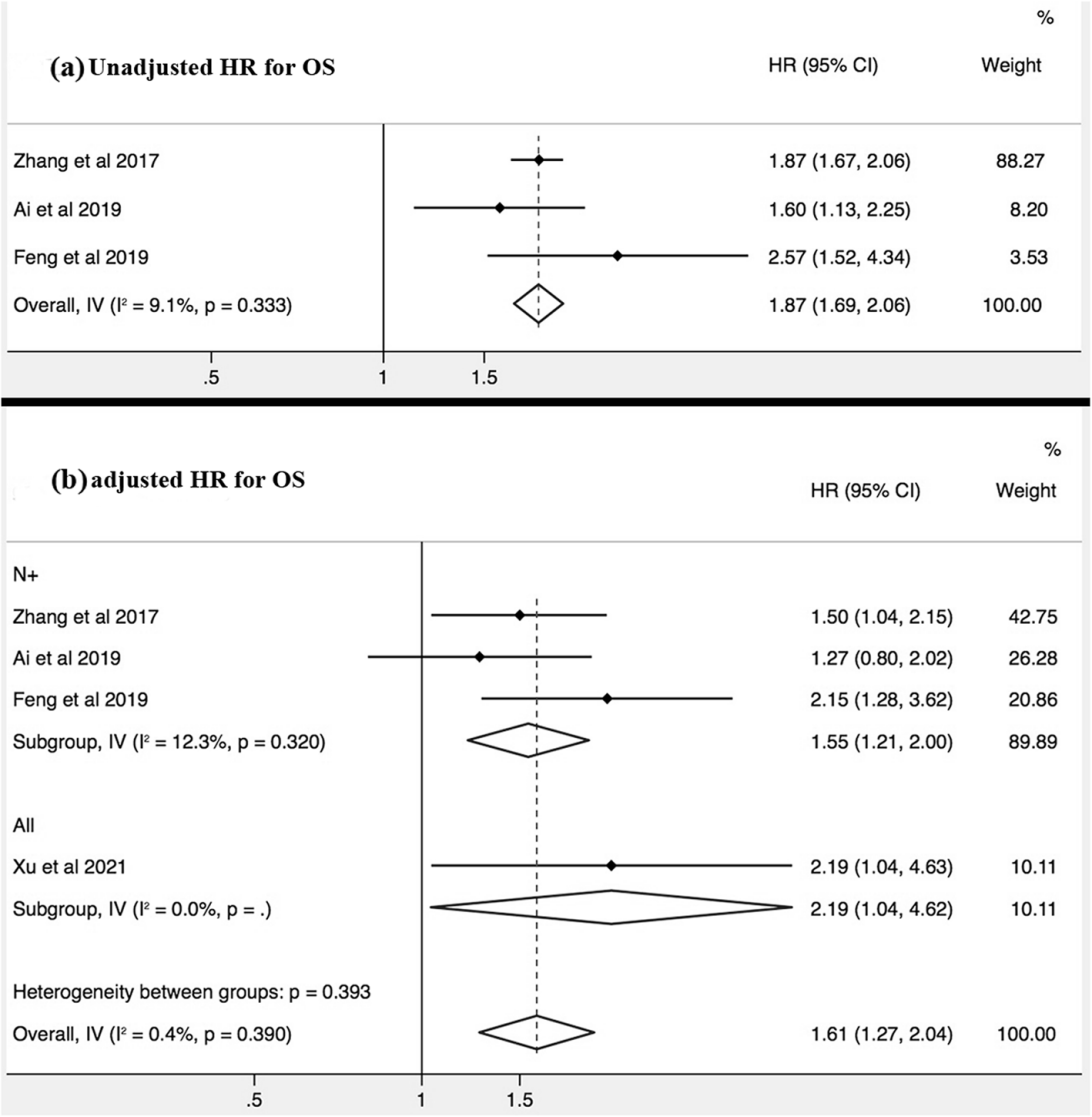
Forest plots of the meta-analysis showing the pooled hazard ratios (HRs) of CNN presence in any nodal group for overall survival (OS). Meta-analysis was performed for the unadjusted HRs (**a**) and adjusted HRs (**b**), respectively

Of the 6 studies that are eligible for meta-analysis, 4 studies performed the survival analysis in patients with metastatic nodes (N+ group), and 2 in all patients (Table 2). The unadjusted and adjusted HRs of CNN were reported for DMFS in 5 and 5 studies respectively, for DFS in 3 and 4 studies, respectively, and for OS in 3 and 4 studies, respectively (Figs. 2,3,4).

**Fig. 5:**
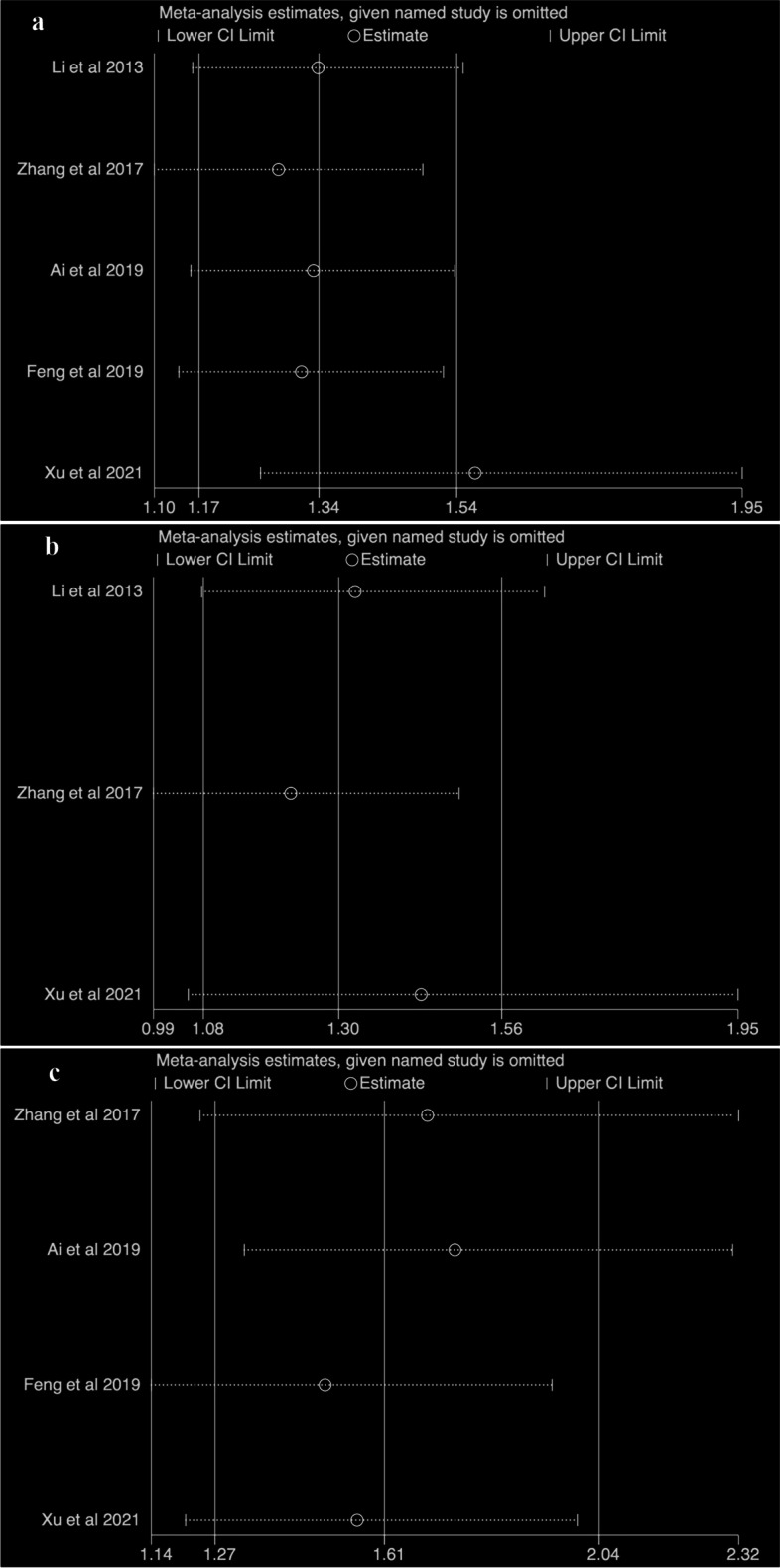
Sensitivity analysis showing the association between the adjusted hazard ratios (HRs) of CNN presence in any nodal group and the distant metastases-free survival (**a**), disease-free survival (**b**) and overall survival (**c**)

The pooled unadjusted HRs showed that the presence of CNN predicted poor DMFS (HR = 1.89, 95%CI = 1.72 – 2.08, I^2^ = 44.8%, *p* = 0.123) (Fig. 2), DFS (HR = 1.57, 95%CI = 1.08 – 2.26, I^2^ = 71.1%, *p* = 0.063) (Fig. 3), and OS (HR = 1.87, 95%CI = 1.69 – 2.06, I^2^ = 9.1%, *p* = 0.333) (Fig. 4). The pooled adjusted HRs also showed the consistent results for DMFS (HR = 1.34, 95%CI = 1.17 – 1.54, I^2^ = 0.0%, *p* = 0.426) (Fig. 2), DFS (HR = 1.30, 95%CI = 1.08 – 1.56, I^2^ = 12.7%, *p* = 0.318) (Fig. 3), and OS (HR = 1.61, 95%CI = 1.27 – 2.04, I^2^ = 0.4%, *p* = 0.390) (Fig. 4). The sensitivity tests of the unadjusted and adjusted HRs for the survival endpoints in the meta-analysis are shown in Fig. 5. Subgroup meta-analysis was performed for the adjusted HRs to further evaluate the prognostic value of CNN presence/absence in patients with metastatic nodes (N+ group). Results showed that the presence of CNN predicted poor DMFS (HR = 1.56, 95%CI = 1.25 – 1.95, I^2^ = 0.0%, *p* = 0.837) (Fig. 2), DFS (HR = 1.43, 95%CI = 1.05 – 1.95, I^2^ = 40.1%, *p* = 0.196) (Fig. 3), and OS (HR = 1.55, 95%CI = 1.21 – 2.00, I^2^ = 12.3%, *p* = 0.320) (Fig. 4).

The Begg’s and Egger’s tests showed that no potential publication bias were observed in the meta-analysis (Begg’s, *p* = 0.211 to >0.999; Egger’s test, *p* =0.132 to 0.905) except for that of the unadjusted HRs for DMFS (Egger’s test, *p* = 0.014).

### Prognostic values of other CNN patterns

Tang et al [25] evaluated the prognostic value of the presence of CNN in the retropharyngeal nodes (RPNs) in patients with metastatic nodes (N+ group) showing the presence of CNN independently predicted poor DMFS (HR = 1.75, 95%CI =1.10 – 2.79), and DFS (HR = 1.80, 95%CI =1.21 – 2.65). Zhang et al [26] classified patients with nodes into 3 grades of necrosis (grade 0: no necrotic area; grade 1: any node with necrotic area of ≤33%; and grade 2: any node with necrotic area of > 33%) and added the necrosis grades as the continuous variable to the survival analysis. Results showed that patients with higher necrosis grades independently predicted poorer DMFS (HR = 1.36, 95%CI =1.14 – 1.63), DFS (HR = 1.38, 95%CI =1.21 – 1.59), and OS (HR = 1.36, 95%CI =1.13 – 2.45). Xie et al [27] reported patients with bilateral CNN independently predicted poorer DMFS (HR = 2.10, 95%CI =1.10 – 2.40) compared to those with unilateral CNN or without CNN. The study conducted by Ai et al. [23] also quantitatively evaluated the total CNN volume and necrosis% showing necrosis%, but not the total CNN volume, was a factor for predicting DMFS (HR = 3.03, 95%CI =1.242–7.397) and OS (HR = 3.09, 95%CI =1.482–6.431); while necrosis% was not an independent factor to predict outcome when other confounding factors were added to the multivariate analysis.

## Discussion

The current review article systematically investigated the studies that evaluated the prognostic values of CNN in patients with NPC, and performed meta-analysis to assess the pooled HRs of CNN presence in any nodal group for DMFS, DFS and OS in 4359 patients. Although over 20 studies have investigated the prognostic value of CNN in patients with NPC, more than half of these studies were excluded for meta-analysis due to insufficient information reported. According the NOS assessment, all eligible studies were scored from 7-9, indicating the data extracted from the studies are reliable. Results from the meta-analysis in 6 studies showed that the CNN presence in any nodal group predicted poor DMFS, DFS, and OS with the pooled adjusted HRs ranging from 1.34 to 1.61 when analysis included all patients, and from 1.43 to 1.56 when analysis only included patients with metastatic nodes (N+ group). Four eligible studies evaluated the prognostic values of five different CNN patterns, which also showed the CNN is a negative factor for outcome.

With the introduction of IMRT to NPC, primary tumour has been well controlled with a local relapse rate of less than 10%, but distant metastases remain problematic and are now the main cause of mortality [40, 41]. More and more studies have found that the presence of nodal metastases is one of the key factors that predict patients at risk of distant metastases. These studies have gone further to investigate the prognostic values of nodal characteristics in detail [42–46]. Necrosis is easily observed in metastatic nodes from NPC but very rarely in primary tumour (about 2%) on the pre-treatment staging MRI [34]. Tang et al [25] reported that CNN was observed in 13% patients with metastatic retropharyngeal nodes, and the current study showed that the incidence of CNN rate was about 42% in patients with metastatic nodes. In the current study, meta-analysis was firstly performed for the unadjusted HRs of CNN, and the results showed that the presence of CNN in any nodal group indeed was a factor to predict poor outcome endpoints. This is expected because necrosis in the pre-treatment malignant tissues commonly indicates the hypoxia of the tissues, and previous studies have also shown that hypoxic malignant tissues are resistant to the treatment [7, 8]. Additionally, meta-analysis was further performed for the adjusted HRs of CNN, and the results indicated that the presence of CNN in any nodal group remained strong to independently predict outcome endpoints after adjusting the other confounding factors.

It is worthy to note that we included five studies conducted by the same institution [19, 20, 25–27]. Two of them evaluated the prognostic value of the presence of CNN in any nodal groups in two separate populations and included in the meta-analysis; and three further evaluated prognostic values of three different CNN patterns, respectively, in the overlapped population. Tang [25] showed patients with CNN observed in the metastatic retropharyngeal nodes had worse outcome compared to those with metastatic retropharyngeal nodes but no CNN. Two studies further qualitatively evaluated the role of CNN burden showing that patients who had grade 2 CNN (with a necrotic area over 33% in any node) [26] or with bilateral CNN [27] had poor outcome. Furthermore, a quantitative analysis [23] showed that necrosis% was a factor that predicted poor outcome. These results indicated the high CNN burden in the nodes may reflect the severe hypoxia in the tumour, thus predicting the high risk of disease recurrence. However, whether the grade of CNN burden was an independent predictor for outcome is still unclear as the multivariate analysis in these studies showed conflicting results while meta-analysis was unlikely to be performed due to the great heterogeneity in the CNN patterns analysed in these studies.

Some studies have proposed their modified criteria by incorporating the CNN to the current staging system showing the improvement in the prognostic performance [10, 21, 22]. However, the differences in the pooled HRs of the CNN and extranodal extension (ENE) reported in a previous meta-analysis (the pooled HRs of the ambiguous ENE ranged from 2.62-3.14) [46]indicate that the modification for the cancer staging system should take into account the role of other nodal characteristics. Although the present study could not answer the question whether and how CNN should be included in the current staging system, our results indicate that the inclusion of CNN is critical in the future studies aiming to investigate the prognostic values of nodal characteristics in NPC.

This study has some limitations that may result in the inherent heterogeneity and the publication bias. First, all studies included in this systematic review were retrospective studies. Due to the retrospective nature, the concerns regarding the risk of bias in the included studies could not be avoid. Second, the unadjusted HR and 95%CI of HRs in one study was extracted and calculated from the survival curves [20]; Third, the potential reasons that resulted in the heterogeneity of the meta-analysis was not able to be analysed due to the limited numbers of the eligible studies; Fourth, the publication bias could not be excluded as studies with positive results were more likely to be accepted for publication; Furthermore, this review article did not include studies that evaluated the prognostic value of CNN showing on the pre-treatment PET/CT. However, PET/CT is routinely performed to evaluate distant metastases rather than CNN in patients with NPC in clinical practice.

## Conclusion

Results from the current study showed that the presence of CNN in any nodal group observed on the pre-treatment staging MRI is a negative factor for DMFS, DFS, and OS in patients with NPC. Although it remains to be defined whether and how CNN should be included in the staging system, the inclusion of CNN is critical in the future survival analysis in NPC.

## Data Availability

Not applicable.

## Abbreviations

95%CI: 95% Confidence interval
CNN: Cervical nodal necrosis
CT: Computed tomography
DFS: Disease free survival
DMFS: Distant metastases free survival
HR: Hazard ratio
MRI: Magnetic resonance imaging
NPC: Nasopharyngeal carcinoma
NOS: Newcastle-Ottawa-Scale
OS: Overall survival
